# Meta-transcriptomics reveals a diverse antibiotic resistance gene pool in avian microbiomes

**DOI:** 10.1186/s12915-019-0649-1

**Published:** 2019-04-08

**Authors:** Vanessa R. Marcelino, Michelle Wille, Aeron C. Hurt, Daniel González-Acuña, Marcel Klaassen, Timothy E. Schlub, John-Sebastian Eden, Mang Shi, Jonathan R. Iredell, Tania C. Sorrell, Edward C. Holmes

**Affiliations:** 1 Marie Bashir Institute for Infectious Diseases and Biosecurity and Sydney Medical School, The University of Sydney, Sydney, NSW 2006 Australia; 2 Westmead Institute for Medical Research, Westmead, NSW 2145 Australia; 3 School of Life & Environmental Sciences, Charles Perkins Centre, The University of Sydney, Sydney, NSW 2006 Australia; 4 WHO Collaborating Centre for Reference and Research on Influenza, at The Peter Doherty Institute for Infection and Immunity, Melbourne, VIC 3000 Australia; 5 Laboratorio de Parásitos y Enfermedades de Fauna Silvestre, Facultad de Ciencias Veterinarias, Universidad de Concepción, 3349001 Concepción, Chile; 6 Centre for Integrative Ecology, School of Life and Environmental Sciences, Deakin University, Geelong, VIC 3216 Australia; 7 Faculty of Medicine and Health, Sydney School of Public Health, The University of Sydney, Sydney, NSW 2006 Australia

**Keywords:** Meta-transcriptomics, Microbiome, Birds, Resistome, Antimicrobial resistance, Wastewater

## Abstract

**Background:**

Antibiotic resistance is rendering common bacterial infections untreatable. Wildlife can incorporate and disperse antibiotic-resistant bacteria in the environment, such as water systems, which in turn serve as reservoirs of resistance genes for human pathogens. Anthropogenic activity may contribute to the spread of bacterial resistance cycling through natural environments, including through the release of human waste, as sewage treatment only partially removes antibiotic-resistant bacteria. However, empirical data supporting these effects are currently limited. Here we used bulk RNA-sequencing (meta-transcriptomics) to assess the diversity and expression levels of functionally viable resistance genes in the gut microbiome of birds with aquatic habits in diverse locations.

**Results:**

We found antibiotic resistance genes in birds from all localities, from penguins in Antarctica to ducks in a wastewater treatment plant in Australia. Comparative analysis revealed that birds feeding at the wastewater treatment plant carried the greatest resistance gene burden, including genes typically associated with multidrug resistance plasmids as the *aac(6)-Ib-cr* gene. Differences in resistance gene burden also reflected aspects of bird ecology, taxonomy, and microbial function. Notably, ducks, which feed by dabbling, carried a higher abundance and diversity of resistance genes than turnstones, avocets, and penguins, which usually prey on more pristine waters.

**Conclusions:**

These transcriptome data suggest that human waste, even if it undergoes treatment, might contribute to the spread of antibiotic resistance genes to the wild. Differences in microbiome functioning across different bird lineages may also play a role in the antibiotic resistance burden carried by wild birds. In summary, we reveal the complex factors explaining the distribution of resistance genes and their exchange routes between humans and wildlife, and show that meta-transcriptomics is a valuable tool to access functional resistance genes in whole microbial communities.

**Electronic supplementary material:**

The online version of this article (10.1186/s12915-019-0649-1) contains supplementary material, which is available to authorized users.

## Background

Tons of antibiotics are used annually in clinical and agricultural settings worldwide. Food animals alone consumed over 130,000 tons of antibiotics in 2013 [1], and antibiotic usage by humans increased 65% between 2000 and 2015, reaching 34.8 billion defined daily doses [2]. The resulting proliferation and spread of bacteria that are resistant to antibiotics poses a major health and economic threat [3]. Genes for the production of antibiotics and antibiotic resistance determinants are found naturally in some microbial species and their presence in the environment is not necessarily an indication of human impact [4, 5]. However, the use of antibiotics in clinical and agricultural settings selects for bacteria carrying resistance genes. When these genes are encoded in mobile elements, such as plasmids and conjugative transposons, they can be readily transmitted via horizontal gene transfer between environmental bacteria and human pathogens (i.e., acquired resistance genes). Multiple resistance genes can be present in a single mobile element, and the spread of plasmid-borne resistance has jeopardized the efficacy of many antibiotics, including β-lactam drugs used as a last resort [6, 7].

Both the environment and wildlife are major sources and reservoirs of resistance gene diversity [8, 9]. The ecological niches and behavior of birds make them particularly likely to transport antibiotic-resistant bacteria. Migrating bird species transport pathogens which may contain antibiotic resistance genes across large distances [10, 11]. Birds also serve as sensitive bioindicators of environmental contamination with antibiotic-resistant bacteria [10, 12–16]. For instance, ESBL-producing *Escherichia coli* were found to occur over three times more frequently in gulls than in humans in the same region [15]. Bacteria resistant to β-lactam and tetracycline drugs are commonly found in the gut microbiome of birds, especially in scavenging and aquatic species, such as waterfowl, gulls, and waders [10, 12, 14, 17–20]. Aquatic bird species likely acquire these genes through contact with contaminated water. Human sewage is enriched in antibiotic-resistant bacteria, which are only partially removed during the water treatment process [21–26]. Birds in contact with wastewater treatment influents or effluents could therefore be at increased risk of acquiring these genes, although empirical data to support this idea are scarce [8].

While the majority of studies on birds were based on bacteria cultured in vitro, the development of culture-independent sequencing techniques has substantially expanded our knowledge of the environmental reservoir of resistance genes [7, 9, 27–32]. Among these techniques, sequencing the entire set of transcribed (i.e., expressed) genes via “meta-transcriptomics” has rarely been used in the context of antibiotic resistance, despite its advantages. In particular, use of meta-transcriptomics allows data to be obtained from the entire microbial community, with a focus on functionally active genes. This is important because genetic material is a metabolic burden and genes that are not essential tend to be lost [33–36]. In the absence of selection pressure exerted by antibiotics, it is likely that resistance genes are regularly lost by bacteria, either by large deletions or gradual deactivation (erosion). Other high-throughput techniques, such as DNA-based metagenomics, cannot distinguish recently deactivated resistance genes from their functional relatives. An alternative is to clone inserts from environmental strains into cultivable vectors (e.g., *E. coli*), select for resistance in vitro, and then sequence their genomes (e.g., [17, 22]). However, this approach can result in bias towards genes present in organisms closely related to the cloning vector [7]. Meta-transcriptomics does not have this limitation as the transcripts of all microorganisms are assessed using bulk RNA sequencing. To our knowledge, only two studies have used meta-transcriptomics to report on the presence of resistance genes that are functionally active under natural conditions in human and environmental samples [37, 38].

We used meta-transcriptomics to assess the diversity and abundance of antibiotic resistance genes transcribed in the microbiome of water birds of Australia and penguins in Antarctica. Birds were sampled across a range of habitats, from remote locations in Antarctica and Australia, beaches in Melbourne, the second largest city in Australia, to the ponds of a wastewater treatment plant (WWTP) processing half of Melbourne’s sewage. We specifically tested whether ducks from the WWTP harbor a higher diversity and abundance of acquired resistance genes, as might be expected given their exposure to partially treated human waste. Additionally, we explored possible associations between resistance gene burden and intrinsic bird traits such as feeding behavior, taxonomic order, and gut functional profile (expression of metabolic pathways by the microbiome).

## Results

**Fig. 1:**
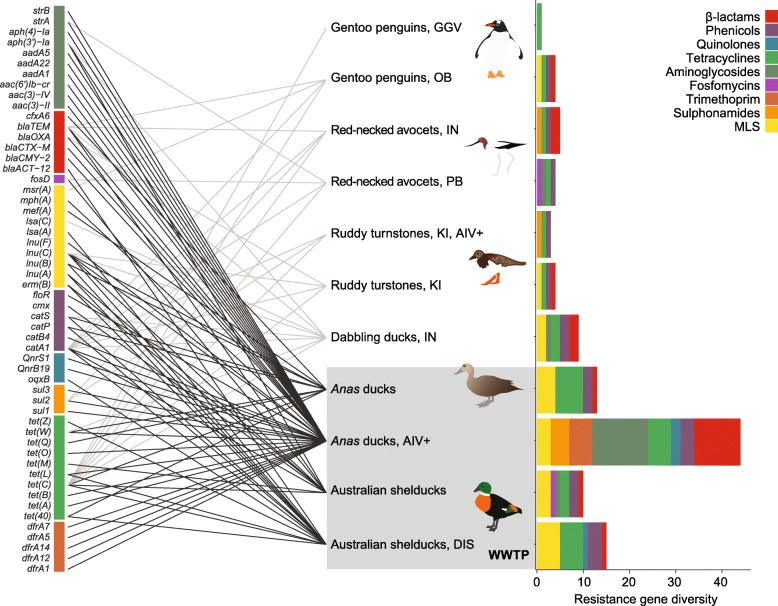
Antibiotic resistance genes expressed in the microbiome of wild birds. The graph on the right shows the diversity of resistance genes observed in each library (containing a pool of 10 individual birds each), colored by the drug class to which these genes confer resistance. Closely related gene variants were merged into one category (see Table S2) for representation on the left side of the figure. Lines link genes to the libraries where they were found, and dark lines indicate the genes observed in the wastewater treatment plant (WWTP) in Melbourne, Australia. PB = Western Port Bay, Melbourne area, Australia; KI = King Island, Bass Strait, Australia; IN = Innamincka reserve, Australia; OB = O’Higgins Base, Antarctica; GGV = Gabriel González Videla Base, Antarctica. Libraries of birds infected with avian influenza virus are indicated with “AIV+,” and the library of diseased birds is indicated with “DIS.” MLS = Macrolides, Lincosamide and Streptogramin B resistance. Bird drawings: M. Wille

Microbiome samples from 110 birds, grouped into 11 libraries (Additional file 1: Table S1), contained transcripts corresponding to 81 unique antibiotic resistance genes associated with phenotypic resistance to nine classes of antibiotics (Fig. 1, Additional file 1: Table S2). These results only include acquired resistance genes, which are most commonly spread among bacteria via mobile genetic elements, and do not include resistance mediated by chromosomal mutations (e.g., in housekeeping genes). Resistance to tetracyclines and phenicols (chloramphenicol and florfenicol) was present in samples from all bird orders and in all locations, except for one site in Antarctica where phenicol resistance was not detected.

### Anthropogenic impact

**Fig. 2:**
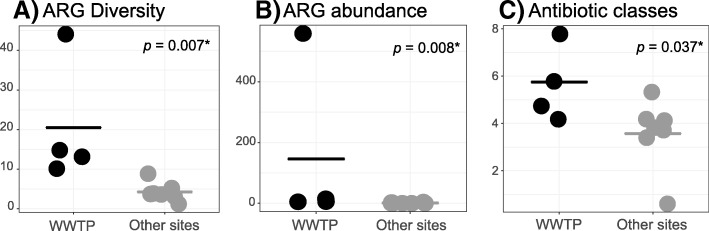
Diversity and abundance of antibiotic resistance genes (ARG) in birds foraging in a wastewater treatment plant (WWTP) compared with birds from other sites in Australia and Antarctica. Each dot represents a meta-transcriptome library (constructed from 10 samples) and cross bars represent mean values. **a** Number of antibiotic resistance genes. **b** Abundance of resistance genes. **c** Number of antibiotic classes. Differences between groups were assessed with a Kruskal-Wallis test and were found to be statistically significant (*p* values < 0.05)

Birds foraging at the partially treated lagoons of a wastewater treatment plant (the last stage of the wastewater treatment process, after aerating and decanting has taken place) had a significantly higher diversity and abundance of antibiotic resistance genes, as well as a significantly higher number of antibiotic classes against which these genes confer resistance (Kruskal-Wallis *p* < 0.05, Fig. 2). For simplicity, we refer to the resistance gene diversity, abundance (i.e., gene expression levels), and number of antibiotic classes to which these genes confer resistance as “resistance gene burden” or “resistance load.” Most notably, ducks (order Anseriformes) foraging at the WWTP harbored 86% of the resistance gene diversity observed, most of which occurred exclusively at the WWTP (Figs. 1 and 2, Additional file 1: Table S2). Pearson’s correlation, Spearman’s correlation, and nested linear regression models showed that the greater resistance gene burden in birds from the WWTP is not a sequencing depth artifact (Additional file 1: Table S3, Additional file 2: Supplementary Materials) [27, 39–51].

One of the libraries contained a much higher abundance of resistance genes (559) than all other libraries (3.3, ± 4.6 std). This library contained ducks infected with avian influenza. To take into account potential confounding variables, we repeated the analyses excluding diseased birds or birds infected with avian influenza (Additional file 2: Figure S1). The results confirm that birds from the WWTP have a higher resistance gene burden regardless of their health status, although more samples would be desirable to test this statistically as the remaining number of libraries precluded statistical analysis.

When only ducks were considered in comparing the effects of wastewater, we observed that those from the WWTP carried more resistance genes than ducks from the remote Innamincka reserve, located in the interior of Australia (Fig. 1, Additional file 1: Table S4). As only one duck library from a pristine site (Innamincka) was available, it was not possible to perform statistical tests; therefore, we present descriptive results: ducks from the Innamincka reserve carried nine resistance genes, fewer than the number observed in any library from the WWTP (average 20.5, ± 15.8 SD). The abundance of these genes was also smallest in ducks from Innamincka (2.9, compared with an average of 146.1, ± 275.2 SD in ducks from the WWTP). The number of antibiotic classes to which these genes confer resistance did not differ substantially between sites (5 antibiotics in birds from Innamincka, compared with 5.7, ± 1.7 in birds from the WWTP).

We also assessed libraries by individual collection localities. Although no statistical analyses were performed given the small number of libraries per collection site, the results graphically show that birds from the WWTP have a higher resistance gene burden than birds from other localities (Additional file 2: Figure S2).

Importantly, an additional PCR-based assessment of the resistance genes in individual birds from two libraries (*n* = 20 samples) confirmed the results obtained using meta-transcriptomics with strong statistical support: we observed 68 resistance gene occurrences (amplifications) in samples from the WWTP and only 12 occurrences in other sites (Kruskal-Wallis *p* = 0.0023, Additional file 2: Figure S3 and Additional file 2: Supplementary Materials).

Samples from gentoo penguins (*Pygoscelia papua*) collected in two localities next to research bases in Antarctica, contained five resistance genes in total, conferring resistance against β-lactams (*bla*_TEM_), tetracyclines (two variants of *tet*(C)), chloramphenicol (*catA1*), and erythromycin (*msr*(A)) (Table S2). The erythromycin-resistance gene, which confers resistance to Macrolides, Lincosamide, and Streptogramin B, was observed in penguins only. Penguins living near the research base with the largest human population (O’Higgins Base) contained more antibiotic resistance genes (four genes: *bla*_TEM_, *msr*(A), *catA1*, and *tet*(C)), than those living next to the more remote Gabriel González Videla Base (one *tet*(C) gene).

### Host traits and functional context

Our sampling design included birds from a range of habitats and species, which will impact their microbiome and possibly their propensity to carry antibiotic resistance genes. Shelducks and *Anas* ducks (Anseriformes) feed by dabbling (filtering water). Turnstones and avocets (Charadriiformes) commonly prey on invertebrates, and penguins (Sphenisciformes) prey on fish. Dabbling ducks live in a range of habitats, including nutrient-rich and heavily altered environments. The majority of ducks analyzed here were sampled at the WWTP: 40 samples (4 libraries) at the WWTP and 10 samples (1 library) in a pristine site. Turnstones and penguins on the other hand live in pristine habitats. Host taxonomic order therefore serves as a proxy for the ecology of the birds analyzed here. Our results indicated that ducks contained the greatest diversity and abundance of resistance genes, while penguins contained the lowest (Fig. 1 and Additional file 2: Figure S4).

Host ecology is intrinsically linked to microbiome function. By investigating how microbiomes functionally differ among bird orders and collection sites, we can gain insights into why some hosts harbor more resistance genes than others. We characterized the metabolic pathways expressed by the microbial community (that is, their functional profile, Table S5). Some of the metabolic pathways observed were produced by common human pathogens (e.g., *E. coli*), but a large proportion of the metabolic products (91%) could not be associated with particular bacterial genera (Additional file 1: Table S5). Compared with the human gut, the microbiome of wild animals is far less characterized, and it is expected that several bacterial species were undetected. Principal coordinate analyses showed that ducks (from Innamincka reserve and from the WWTP) have a distinct microbial metabolism (i.e., set of metabolic pathways) when compared with birds from other sites (Additional file 2: Figure S5). We statistically assessed the distinctiveness of functional profiles between sites and bird orders using Random forest analysis, a machine learning approach based on classification trees that has a high discriminating power for use in microbial ecology [52]. This analysis revealed a clear distinction (zero out-of-bag classification error) in the functional profiles between birds from the WWTP and other sites, and between Anseriformes and the two other bird orders that comprised the data set (Charadriiformes and Sphenisciformes; Additional file 1: Table S6).

We have also observed that the abundance of resistance genes correlates with the number of mRNA reads attributed to the microbial community (i.e., after removing host reads), at least when using one of the correlation tests performed (Spearman’s correlation, *p* = 0.04, Additional file 2: Supplementary Methods) [27, 39–51]. This correlation suggests that it is possible that birds with a higher resistance gene burden also have a higher abundance of gut bacteria.

The bird microbiome, and consequently its functional profile, can also be affected by avian pathogens [53]. We sampled birds with avian influenza virus infection and Newcastle disease symptoms; potential associations between these infections and antibiotic resistance are discussed in the Additional file 2: Supplementary Results and Discussion [53–57].

## Discussion

This study shows that clinically important and functional antibiotic resistance genes are widespread, even in birds from areas as remote as Antarctica, and that the resistance gene load is significantly higher in birds living in the lagoons of a wastewater treatment facility. Meta-transcriptomic data are highly informative, and even though the number of libraries was relatively small, it was possible to perform meaningful comparisons between birds from different localities that will guide future studies. Although resistance genes can be found in natural environments regardless of human influence [4, 5], our results suggest that contact with human waste—even if it goes through sewage treatment—appears to impact the acquisition of antibiotic resistance genes by avian wildlife.

The resistance genes observed here encompass the three major resistance mechanisms of relevance to human infection: (i) drug inactivation, (ii) reduced influx of antibiotics into bacterial cells or increased efflux from cells, and (iii) alteration in, or overexpression of, the antibiotic target [7, 58]. The observed resistance genes conferred resistance against nine classes of antibiotics (Fig. 1). This number is slightly higher than the six classes of antibiotic resistance observed in humans, pigs, sponges, and environmental samples in another study which used meta-transcriptomics [37]. Among the most common were genes conferring resistance to β-lactam drugs, which form one of the oldest and most widely used antibiotic classes. Genes conferring resistance to aminoglycosides and tetracyclines were also common, in agreement with studies reporting these genes in human-impacted soils and sewage [22, 27, 29, 31].

Some of the resistance genes observed are particularly concerning for public health. *bla*_CTX-M_ genes, observed exclusively in birds from the WWTP, play a key role in widely disseminated and highly resistant strains of *E. coli* and *Klebsiella pneumoniae* [59]. A fosfomycin resistance gene (*fosD*) was found in birds from metropolitan Melbourne (WWTP and Western Port Bay). Fosfomycin was discovered over 40 years ago; it is uncommonly used in humans, but the low resistance levels against this drug have led to a renewed interest in its therapeutic use [60]. One of the bird libraries from the WWTP contained a florfenicol resistance gene, which was first observed in *Salmonella typhimurium* [61]. Florfenicol is restricted to livestock and veterinary use. It is possible that the presence of this gene is due to the administration of florfenicol to pets and wildlife within the WWTP catchment range. The florfenicol gene has been observed co-located with other resistance genes in integrons and plasmids [61, 62]. It is therefore also possible that this gene is present in the WWTP due to co-selection with other genes. We also found resistance against chemically synthesized antibiotic classes, such as quinolones and sulphonamides, which are not expected to be widespread in the environment (unlike naturally produced antibiotics such as penicillin, which is derived from fungi). Quinolone drugs can persist in the environment for long periods [63], and despite being a synthetic drug, the origins of quinolone resistance have been traced back to aquatic bacterial species [64]. Therefore, it is perhaps unsurprising that these genes are found in birds with aquatic behavior (also reported in [19]). It is noteworthy, however, that quinolone resistance was only observed in birds near the WWTP, suggesting that these genes most likely derive from bacteria of human origin. One of the WWTP libraries also contained the *aac(6)-Ib-cr* gene (100% identity with clinical isolates), which confers resistance to quinolones and aminoglycosides and is often localized in multidrug resistance plasmids. First reported in Shanghai in 2003, this gene has already been found in several parts of the world, including in a recent report of multidrug-resistant *Salmonella* in Australia [7, 65, 66].

The pool of antibiotic resistance genes is directly linked to microbial species composition and environmental conditions [67, 68]. Some members of the microbiome are more prone to carry resistance genes than others—β-lactam resistance, for example, is more common in Actinobacteria than in other phyla [68]. Likewise, the resistance gene burden among humans is influenced by their enterotypes [69]. The possible effects of microbiome composition prevent implicating human impact as the sole cause of high resistance gene burden in birds from the WWTP. In fact, it is reasonable to assume that microbial community composition differs across localities and bird species, given their distinct ecological niche. Penguins and avocets hunt small aquatic animals, while ducks filter water and sediments to trap plant and animal material. It is possible that ducks ingest large numbers of bacteria while dabbling. In addition, birds may have historical-evolutionary associations with particular microbial species, resulting in a distinct microbiome composition and functioning across avian taxonomic groups. Indeed, a metabarcoding study showed that bird taxonomy explained most of the compositional variation in their microbiomes [70]. Our functional analyses also suggest that the metabolism of microbial communities in different bird orders is distinct. The microbiome of Anseriformes (ducks) expressed genes encoding significantly different metabolic pathways than other birds, while there was no clear distinction among Charadriiformes and Sphenisciformes (Additional file 1: Table S6 and Additional file 2: Figure S5). It is plausible that the comparatively high resistance gene expression in ducks from the WWTP is influenced by their distinct microbiome, which in turn reflects their ecological niche and established host-microbe associations. In this scenario, bird traits modulate the human impact on the spread of resistance genes, amplifying or diminishing it according to their habits and the composition of their microbial communities. Further studies are necessary to test this hypothesis and disentangle the effects of bird traits (including microbiome composition) and human impact on resistance gene burden.

Migratory birds are of particular concern as they might spread antibiotic resistance across large geographic distances in the same way that they disperse pathogens [9, 11, 71, 72]. There are significant differences in the gut microbiomes of migratory and resident red-necked stints (*Calidris ruficolis*) and curlew sandpipers (*Calidris furringea*), although these differences may be temporary [73, 74]. Ruddy turnstones have a remarkable migration habit, traveling between breeding areas in Siberia to non-breeding sites in Australia via East-Asia, potentially acquiring and distributing resistant bacteria along the way. The turnstones analyzed here carried resistance genes against several antibiotic classes, but the diversity of genes within those classes was much smaller than in birds at the WWTP (Fig. 1). *Anas* ducks travel hundreds of kilometers within Australia [75]. It is possible that ducks from the Innamincka reserve have been in sites of high human impact previously, resulting in the higher load of resistance genes when compared with other birds from remote areas. It is also plausible that ducks acquire resistant bacteria due to their feeding behavior and the composition of their gut microbiome.

Despite their isolation, we found genes conferring resistance against four antibiotic classes in penguins from Antarctica. Previous studies of antibiotic resistance in penguins have produced contradictory results. In one, various tetracycline-resistant bacteria were isolated from the cloaca of penguins [76], while in another high levels of resistance against multiple antibiotics were detected in penguin droppings [77]. However, other studies have reported that antibiotic-resistant bacteria are rare in these animals [24, 78, 79]. It is possible that penguins acquire resistance genes from migratory fish and other prey or animals with which they interact. As antibiotics are naturally produced by bacteria, it is also possible that the resistance genes observed in the penguin microbiome occur in the environment regardless of human influence. The possibility of some cross-library and/or environmental contamination cannot be completely excluded. Nevertheless, the bona fide influence of human activity is supported by the larger number of resistance genes adjacent to the more populated O’Higgins Base compared with the much smaller González Videla Base. Additionally, previous research has documented higher antibiotic resistance levels near research facilities compared to more pristine sites in Antarctica [24, 77]. Human impacts, including increasing research activities, tourism, and limited sewage treatment [80, 81], are therefore the most likely explanation for the presence of antibiotic resistance in Antarctic penguins.

The bird microbiome expressed resistance against nine classes of antibiotics, even though we putatively enriched libraries with resistant bacterial strains using only two classes of antibiotics in the collection media (aminoglycoside and β-lactams, see “Methods”). Acquired (horizontally transferred) resistance genes can be constitutively expressed, in which case the presence of their transcripts is expected even without antibiotic exposure. It is also possible that these resistance genes were acting against antibiotics present in the environment and/or that these genes are co-transmitted with others that have functions in addition to antibiotic resistance (e.g., metal resistance, [82]). Meta-transcriptomic studies necessarily rely on reference databases, which limits the discovery of novel resistance genes [30], and the database used here (ResFinder [83]) does not include resistance that arises through de novo mutation in the bacterial genome (which would increase the detection of false positives). Therefore, although we chose to assess acquired resistance genes only, it is the genes residing on mobile elements that pose the greatest public health risk, as they can be transferred easily between bacteria [84]. Considering our rather conservative analyses (see “Methods”), it is possible that we have underestimated the presence of some resistance genes that were not expressed or were expressed at low abundance. Finally, variables related to the ecology, geographic distribution, and composition of the microbial communities of the birds likely influence resistance gene burden. Results based on individual collection sites and bird taxonomic group suggest that these variables are unlikely to change the conclusion that birds from the WWTP carry the highest diversity and abundance of resistance genes, but more replicates are required to determine the relative contribution of the multiple factors influencing resistance gene diversity and abundance.

## Conclusions

We show for the first time that ducks feeding on wastewater are particularly prone to harbor bacteria with transcriptionally viable antibiotic resistance genes. Further studies are warranted to disentangle the underlying causes of the correlation between WWTP and resistance gene burden observed here. Ecological and functional traits are likely intertwined in explaining the higher propensity of ducks to carry antibiotic resistance genes. This study also contributes to the increasing literature reporting widespread antibiotic resistance in birds, even in isolated areas like the Australian outback and Antarctica. For antibiotic-resistant bacteria, aquatic systems are major traffic routes between wildlife and humans [8, 85]. The resistance genes acquired by birds can be re-introduced in the environment, possibly into different water systems (e.g., by migrating ducks) and might re-infect humans directly via contact with contaminated water, or indirectly by the introduction of these genes into the food chain [85]. Investigating the mechanisms that sustain the persistence and cycling of resistance genes in wild populations despite the metabolic burden that these genes impose is a logical next step towards tackling antibiotic resistance.

## Methods

### Sampling

Samples were collected as part of long-term avian influenza virus surveillance studies [86–91]. Ethics approvals, bird capture methods, and sample handling are reported in the Additional file 2: Supplementary Materials. In short, cloacal swabs were collected using a sterile-tipped applicator. Additional oropharyngeal swabs were collected for ruddy turnstones in King Island and merged with their cloacal swabs. Samples were placed in viral transport media (VTM, Brain-heart infusion broth containing 2 × 10^6^ U/L penicillin, 0.2 mg/ml streptomycin, 0.5 mg/ml gentamicin, 500 U/ml amphotericin B, Sigma), kept refrigerated (4–8 °C), and stored at − 80 °C within 8 to10 h of collection, with the exception of samples from turnstones, which were kept refrigerated for up to 7 days after collection before being stored at − 80 °C. VTM is a standard buffer used in avian influenza surveys and has the advantage of killing a portion of non-resistant bacterial strains. This step enriches meta-transcriptome libraries with antibiotic-resistant bacteria and, consequently, increases the sensitivity of the antibiotic resistance survey. Naturally, antibiotic treatment can induce the expression of antibiotic resistance, allowing their detection with meta-transcriptomics. Apart from the abovementioned exceptions with turnstones, all samples were processed in the same manner. The resistance gene burden of turnstones does not differ substantially from other non-WWTP samples (or other Charadriiformes libraries—e.g., Figure 1), and therefore there is no reason to believe that sample processing would impact the conclusions of this study.

All birds in this study were apparently healthy, with the exception of one library constructed from dead and dying shelducks with symptoms of Newcastle disease. Samples were assayed for avian influenza virus as previously described [87]. Samples were collected at sites with different levels of anthropogenic impact (Additional file 2: Supplementary Materials). Birds sampled at the WWTP were found in lagoons composed of partially treated water (the final stage of wastewater treatment).

### RNA-sequencing and data processing

RNA isolation procedures are detailed in Additional file 2: Supplementary Materials. Libraries were composed of 10 conspecific bird samples pooled at equal concentrations. Paired-end sequencing (100 bp) was performed on a HiSeq2500 platform, and the number of reads obtained is reported in Table S1. Low-quality reads, adapters, host reads, and ribosomal RNA were filtered out from the data set (Additional file 2). The commands used to perform quality control can be found in Additional file 3.

### Resistance gene characterization and functional profiling

The ResFinder reference database [83] was used in conjunction with the KMA program [92] (downloaded in December 2017) to identify resistance genes in the meta-transcriptomic data set. The ResFinder database currently contains 2255 resistance genes compiled from published manuscripts and existing databases. KMA was preferred over other alignment tools because it performs well in aligning short reads against highly redundant databases and is able to resolve non-unique read matches by assessing and statistically testing global alignment scores. To minimize the risk of false positives and increase the minimum mapping length allowed, only genes with a mapping coverage greater than 20% were considered in the analyses, all of which had an alignment *p* value < 0.05. The average length of the resistance genes observed was 944 bp—a gene with this length was only considered in the downstream analyses if query reads overlapped by at least 189 bp (20% coverage). This approach is highly conservative because it uses an aligner that yields a minimal number of false positives [92], does not include housekeeping genes (which would increase the occurrence of false positives), and defines resistance genes based on gene fragments (at least 20% of the genes) rather than individual reads. The gene fragments analyzed here are longer than the ones obtained via qPCR (generally 100 bp amplicons), which are widely used in AMR assessments of environmental samples and in diagnostic laboratories. One gene (*bla*_TEM-116_) was observed in all libraries but was removed from the analyses due to its potential contaminant nature [49]. It is possible that the data set contains other laboratory contaminants, but the fact that one of the libraries contained only one resistance gene, and that no other gene (except for *bla*_TEM-116_) was found in all libraries, suggests that contamination is unlikely. Genes conferring resistance to Macrolide, Lincosamide, and Streptogramin B were considered as one antibiotic class (MLS). Absolute read abundances were estimated based on a stably expressed host gene and normalized for gene length (Additional file 2: Supplementary Materials).

The microorganism-based functional profile was inferred with HUMAnN2 [93] (http://huttenhower.sph.harvard.edu/humann2), using the UniRef90 protein database as reference [94].

### Statistical analyses

The number of antibiotic classes to which resistance was found, the diversity (i.e., number of genes), and the abundance of resistance genes in each library were classified into two bins (“WWTP” and “Other,” Fig. 2). Differences between WWTP and other sites were tested with a Kruskal-Wallis test using the native *stats* R package (R Core Team [95]). Differences were considered significant when *p* values were < 0.05. The R script to perform these statistical analyses and produce Fig. 2 can be found in Additional file 4.

The higher diversity of resistance genes in libraries from the WWTP was validated with a PCR-based approach targeting ten resistance genes in individual birds from two libraries (*n* = 20, Additional file 2: Supplementary Materials). Differences in the number of genes that amplified per sample between WWTP and a pristine site were statistically assessed with a Kruskal-Wallis test (R script in Additional file 4, Results in Additional file 2: Figure S3).

The potential of uneven sequencing depth to confound our results was tested in two ways: first, using Pearson’s and Spearman’s correlation to investigate whether library size correlates with antibiotic resistance gene diversity or abundance, and second, by using nested linear regression models to assess the impact of adding sequencing depth as a co-variate. The tests are detailed in Additional file 2: Supplementary Materials, and the R script is available in Additional file 4. These tests show no evidence of library size acting as a confounder.

We also assessed the resistance gene burden in subsets of the data: (i) using only healthy birds and birds that tested negative for avian influenza (Additional file 2: Figure S1), (ii) in individual sampling sites (Additional file 2: Figure S2), and (iii) across bird orders (Additional file 2: Figure S4). In these cases, however, the sampling number precluded statistical tests.

Differences in expression of microbial metabolic pathways between sites and bird orders were visually assessed with principal coordinate analysis, based on a Euclidean distance matrix, with the R package *ape* [96]. To investigate the association between bird taxonomic order and functional profile statistically, Random forest analyses were performed using 1000 trees, with the *randomForest* R package [97]. The R script for these analyses are provided in Additional file 4.

## Additional files

Additional file 1: **Table S1.** Libraries and metadata. **Table S2.** Antibiotic resistance genes observed in bird meta-transcriptomes. **Table S3.** Confounder tests to account for sequence depth. **Table S4.** Antibiotic resistance gene burden in ducks from the WWTP and an isolated nature reserve. **Table S5.** Contribution of the bird microbiome members to the expression of metabolic pathways. **Table S6.** Random Forest classification based on microbial pathways. **Table S7.** Comparable libraries from healthy birds that were positive or negative for avian influenza virus (A) and healthy or diseased birds with symptoms of Newcastle disease (B), and their corresponding resistance gene burden. **Table S8.** Antibiotic resistance genes successfully amplified with PCR. (XLSX 125 kb)

Additional file 2: **Figure S1.** Results considering only healthy birds and those not infected with avian influenza virus. **Figure S2.** Distribution of antimicrobial resistance genes across localities. **Figure S3.** PCR analyses utilizing individual birds confirm that those from the WWTP harbor a higher diversity of antibiotic resistance genes. **Figure S4.** Distribution of antibiotic resistance genes across bird orders. **Figure S5.** Principal Coordinate Analysis of the expression of microbial metabolic pathways. **Figure S6.** No correlation observed between library size and resistance gene burden. **Figure S7.** Resistance gene expression profile varies with avian influenza infection and health status. **Figure S8.** Relationship between number of microbial reads and resistance gene burden. (PDF 5278 kb)

Additional file 3: Detailed report of the settings and commands used for quality filtering, resistance gene characterization, and functional profiling. (TXT 1 kb)

Additional file 4 R scripts to reproduce statistical analyses and graphs. (TXT 9 kb)
