# Supplementary material for: Meta-transcriptomics reveals a diverse antibiotic resistance gene pool in avian microbiomes
Source: BMC Biol. 2019 Apr 8;17:31. doi: 10.1186/s12915-019-0649-1 (PMC6454771; doi:10.1186/s12915-019-0649-1)
Supplement: Supplementary file 2 — Figure S1. Results considering only healthy birds and those not infected with avian influenza virus. Figure S2. Distribution of antimicrobial resistance genes across localities. Figure S3. PCR analyses utilizing individual birds confirm that those from the WWTP harbor a higher diversity of antibiotic resistance genes. Figure S4. Distribution of antibiotic resistance genes across bird orders. Figure S5. Principal Coordinate Analysis of the expression of microbial metabolic pathways. Figure S6. No correlation observed between library size and resistance gene burden. Figure S7. Resistance gene expression profile varies with avian influenza infection and health status. Figure S8. Relationship between number of microbial reads and resistance gene burden. (PDF 5278 kb) [file 12915_2019_649_MOESM2_ESM.pdf]

Additional file 2:
Supplementary Materials and Figures

Meta-transcriptomics reveals a diverse antibiotic resistance gene pool in avian microbiomes.

Vanessa R. Marcelino, Michelle Wille, Aeron C. Hurt, Daniel González-Acuña, Marcel Klaassen, Timothy E. Schlub, John-Sebastian Eden, Mang Shi, Jonathan R. Iredell, Tania C. Sorrell, Edward C. Holmes.

This PDF file includes:

Supplementary Materials
Supplementary Results and Discussion
Figs. S1 to S8

Supplementary Materials

Species selected and study sites

In Australia, two bird species of the order Anseriformes were selected, comprising Australian shelduck (*Tadorna tadornoides*) and Dabbling ducks (*Anas* sp.), as well as two species of Charadriiformes: ruddy turnstone (*Arenaria interpes*) and red-necked avocet (*Recurvirostra novaehollandiae*). Ruddy turnstones and *Anas* ducks are known reservoir hosts for avian influenza A virus. In Antarctica, gentoo penguins (*Pygoscelis papua*) were sampled from both locations (Additional file 1: Table S1). Birds were captured either using a baited funnel walk in traps, cannon nets or mist nets (Australia) or using a hand net (Antarctica). Cloacal samples were collected using a sterile-tipped applicator. Oropharyngeal swabs were also collected for Ruddy turnstones and merged with their cloacal samples. The number of resistance genes in the microbiome of turnstones is similar to the one observed for other birds (e.g. other Charadriiformes), and therefore it is unlikely that this procedure would impact the conclusions of this study. All birds in this study, including the ones infected with avian influenza virus, were apparently healthy, with the exception of one library constructed from dead and dying Australian shelducks. These birds had symptoms of Newcastle Disease (avian avulavirus type 1), such as froth from the mouth and edema in airsacs and lungs. Although the pathogen could not be confirmed with routine Newcastle Disease Virus specific PCR methods, avian avulavirus 1 reads were identified in the meta-transcriptome library of diseased birds.

Samples were collected at sites with different levels of anthropogenic impact. In Australia, birds were collected next to partially treated water (*i.e.* the final stage of wastewater treatment) at the Western Treatment Plant of Melbourne, which is the second largest city in Australia (37°59′11.62″S, 144°39′38.66″E), at Western Port Bay located ~65km from Melbourne (38°13'51.6″S 145°28'43.9″E), at King Island in Bass Strait, Tasmania (39°55′52″S 143°51′02″E), and at Innamincka Regional Reserve, an isolated area in the outback of Australia (27°32′28″S 140°35′47″E). In Antarctica, samples were collected next to Base Bernardo O'Higgins, Kopaitik Island, Rada Covadonga (63°19'S, 57°51'W), and near the Gabriel González Videla Base, Paradise Bay (64°49'S, 62°51'W).

Library preparation and sequencing

RNA was extracted with the MagMax *mir*Vana™ Total RNA isolation Kit (ThermoFisher Scientific) using the automated KingFisher™ Flex Purification System (ThermoFisher Scientific). RNA quality was assessed using the TapeStation 2200 and High Sensitivity RNA reagents (Agilent Genomics, Integrated Sciences). Pools were constructed by selecting the

10 samples with the highest concentration and combining the RNA at equal concentrations, followed by concentrating the pooled RNA using the RNeasy MinElute Cleanup Kit (Qiagen). Libraries were constructed using the TruSeq total RNA library preparation protocol (Illumina) and rRNA was removed using the Ribo-Zero-Gold kit (Illumina). Paired end sequencing (100bp) was performed on a HiSeq2500 platform. Library preparation and sequencing were carried out at the Australian Genome Research Facility (Melbourne). The total number of reads obtained per library are reported in Additional file 1: Table S1.

Data processing

Low quality sequence reads, Illumina adapters and sequences shorter than 50bp were filtered out using Trimmomatic [39] as implemented in KneadData (https://bitbucket.org/biobakery/kneaddata). Host reads were filtered out using the chicken genome as reference (*Gallus gallus* release 90, downloaded from Ensembl), also using KneadData. Ribosomal RNA was removed with SortMeRNA based on 16S, 18S, 23S, 28S, 5S and 5.8S rRNA databases [40-42].

Estimation of absolute gene abundance

Read count normalization significantly affects gene expression analyses [43, 44]. Raw and relative gene expression measurements are not appropriate to compare different gene expression across libraries [45]. One option is to normalise read counts using controls (e.g. stably expressed housekeeping genes) to estimate the absolute gene abundances [45]. Absolute gene abundances were estimated using the formula:

$$\frac{RPK_{AMR}}{RPK_{host}}$$

Equation 1

Where RPK_{AMR} is the antibiotic resistance gene expression in Reads Per Kilobase (RPK) and RPK_{host} is the expression of a stably expressed host gene.

RPK is calculated as:

$$\frac{Nreads}{gene\ length} \times\ 1000$$

Equation 2

Where *Nreads* is the number of mapped reads and *gene length* is the length of the gene in base-pairs.

KMA does not estimate the abundance of genes in RPK, but uses a more accurate approach. Specifically, the abundance of antibiotic resistance genes is estimated within KMA as the total number of nucleotides covering the template divided by the length of the template (this is the *depth* value in Additional file 1: Table S2). In this case, for example, a single read overlapping the end of a 500bp resistance gene by 50bp accounts for 0.1 depth units. If that read was counted in RPKs units, it would be equivalent to 2 RPK (equation 2), but this value is less accurate as only half of the read overlaps the resistance gene. To convert the original KMA depth counts to RPK units, and considering that the reads were 100 bp long, we multiplied depth values by 10. A depth value of 0.1 becomes therefore 1RPK.

We chose the Ribosomal Protein S13 (RPS13), which is known to be stably expressed [46], to calculate RPK_{host}. A host rather than a bacterial gene is desirable for measuring the total gene burden 'per host', rather than 'per bacteria'. Our assessment aims to detect a higher abundance of bacteria harbouring antibiotic resistance genes, rather than a higher abundance of genes per bacterial cell. RPS13 sequences were obtained from reference genomes of each bird order analysed here (GenBank accessions: NW_013185679, NW_008796218 and NW_009650072) and used as reference templates to count host genes. We used the intermediate files from KneadData, where the quality-control has been performed but host reads were not removed. These reads were mapped to the RPS13 references with bowtie2 [47] and the number of mapped reads was obtained with HTSeq [48]. Using the average length of the three reference RPS13 genes (617bp) as gene length, we computed RPK_{host} according to equation 2.

Finally, we calculated normalised gene abundances, which can be used to compare libraries like absolute gene abundances, by dividing RPK_{AMR} by RPK_{host} (equation 1).

Sequencing depth does not confound results

We performed two analyses to test whether unequal sequencing depth across libraries confounds the results. First, we tested whether sequencing depth correlates with resistance gene diversity and abundance using Pearson's and Spearman's correlation and found no evidence for a correlation ($p > 0.05$, Additional file 2: Fig. S6). Second, we used nested linear regressions to test whether adding sequencing depth as a co-variate changes our results (*i.e.* WWTP vs other sites as a predictor of resistance gene burden). The test was performed by fitting a linear regression model with and without library size as a confounding variable (R script available in additional file 4), and comparing the regression coefficient between these models. We found no evidence that sequencing depth affects antibiotic resistance gene burden and no evidence it confounds the relationship between outcomes

and impact of locality (Additional file 1: Table S3). The impact of locality (WWTP and other sites) on resistance gene diversity and abundance remained significant after controlling for sequencing depth. Sequence depth was fairly even across libraries (19,633,686 – 23,825,333 PE reads), and these two independent tests confirm that sequencing depth does not affect our results.

Assessing resistance gene diversity in individual birds using PCR

Each metatranscriptome library contained 10 individual birds from the same site and species. We performed an antibiotic resistance assessment via PCR on individual birds to consider potential variation within libraries and to confirm the results of our metatranscriptome-based approach. Birds from two libraries were analysed: (i) 6_Temperate_Duck_WWTP and (ii) 8_Avocet_YC (Western Port Bay). Ducks from Innamincka reserve are migrating species and because they have potentially been in contact with polluted sites they were not used as a pristine-site representative for the PCR assay. Nucleic acids were extracted using the Qiagen QiaAMP Viral RNA Extraction Kit, with no DNAse treatment. We targeted 10 resistance genes that were (i) observed in at least one of the two libraries via metatranscriptomics, and (ii) that had primers described in the PCR-based resistome study of Zhu et al. 2017 [27]. Specifically, we used the following primer pairs described in Zhu et al. 2017: blaTEM, catA, cfxA, lnuB-01, lnuC, mefA, strA, tetC-01, tetQ, tetW-01. PCR was performed in 25 µL reactions containing Platinum SuperFi Green Master Mix, primers at 0.5µM and 2–7 ng of DNA. The PCR cycle consisted of initial denaturation at 98°C for 30 s, followed by 35 cycles of denaturation at 98°C for 10 s, annealing at 60°C for 10 s and extension at 72°C for 30 s, and a final extension step at 72°C for 5 m. Amplification of resistance genes was assessed in 2% agarose gels.

Seven out of the ten genes tested were successfully amplified. The $\mathit{bla}_{\text{TEM-116}}$ gene is a known laboratory contaminant [49] that was excluded from our metatranscriptome analyses. Indeed, because we amplified bla_{TEM} in the negative control this gene was also excluded from the analyses based on PCR products. The remaining amplified genes are listed in Additional file 1: Table S8. The diversity of antibiotic resistance genes was significantly higher (Kruskal-Wallis p=0.0023) in birds from the WWTP when individual birds are considered (Additional file 2: Fig S3). These results confirm that the metatranscriptome libraries containing 10 individual birds are accurate representations of the resistance gene pool in a given site and bird species.

Bacterial load and antibiotic resistance gene burden

High bacterial abundance in the gut may be associated with an increased potential to harbour resistance genes. We tested for correlations between microbial load (measured as

the number of reads after host and rRNA were filtered out) and resistance gene burden using Spearman's and Pearson's correlation. No significant result (*p*> 0.05) was observed when using Pearson's correlation, but Spearman's correlation revealed a marginally significant correlation (*p* = 0.04) between microbial load and resistance gene abundance and number of antibiotic classes (Additional file 2: Fig. S8). This correlation is possibly driven by a small bacterial load in penguins and one of the turnstone libraries, which have few resistance genes, and a higher bacterial load in some of the duck libraries from the WWTP (Additional file 1: Table S1). This correlation does not affect the conclusions that birds from the WWTP have more resistance genes than birds from other sites.

Data visualisation

Patterns of gene diversity and abundance were visualized with ggplot2 [50]. To produce heatmaps comparing resistance gene expression (Additional file 2: Fig S7), 0.001 was added to all estimated abundances (to exclude zeros and perform log-transformation), the abundances were then $\log_2$-transformed and visualised with Superheat [51].

Supplementary Results and Discussion

Association between resistance genes and avian infectious diseases

The bird microbiome can also be affected by co-infecting pathogens, even in asymptomatic cases of avian influenza virus infection [53]. To test for an association between avian influenza virus (AIV) infection status and the resistance gene load, we compared libraries of conspecific birds collected within the same sampling period and location. Dabbling ducks (*Anas* sp.) that were apparently healthy but infected with AIV (low pathogenic type, clinically asymptomatic in birds) showed a higher number and abundance of resistance genes (Additional file 2: Fig. S7A, Additional file 1: Table S7). Ruddy turnstones (*Arenaria interpres*) infected with AIV had a lower diversity and abundance of resistance genes than their non-infected counterparts, and the overall resistance gene load was much smaller than the one observed in ducks (Additional file 2: Fig. S7A, Additional file 1: Table S7). We additionally tested for an association between resistance gene load and disease state in Australian shelducks (*Tadorna tadornoides*) collected from the same location and sampling expedition. Sick and dying birds (harboring symptoms consistent with Newcastle disease, a severe viral infection) had a higher diversity of resistance genes (15 genes) compared to their healthy counterparts (10 genes), but the abundance levels were similar between the two libraries (4.9 and 5.6 respectively, Additional file 2: Fig. S7B, Additional file 1: Table S7).

The few replicates analyzed here do not allow for a statistical comparison. We can therefore only speculate on the mechanisms influencing the differences in resistance gene expression across avian influenza infection and health status to raise hypothesis for further research. It is possible that similar factors may influence both resistance and viral acquisition and that the observed differences in resistance gene burden are casual rather than a causal effect of the viral infection. It is also possible that bacteria harboring antibiotic resistance have a negative impact on host health. Studies have shown an interconnection between resistance to antibiotics and endurance to host immune system in bacteria [54-57]. While we cannot derive this conclusion from our results, it is conceivable that a higher burden of resistant bacteria leads to some level of immune susceptibility, which results in birds being more susceptible to viral diseases. More studies are warranted to test whether the spread of antibiotic resistance impacts wildlife health and biodiversity loss.

Supplementary Figures

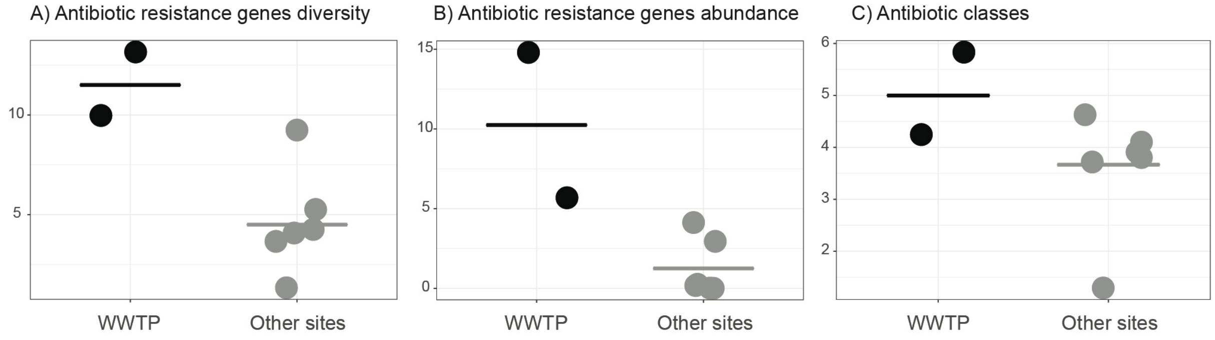

Figure S1. Distribution of antibiotic resistance genes in birds foraging in a wastewater treatment plant (WWTP) compared to birds from other sites in Australia and Antarctica, considering only healthy birds and those not infected with avian influenza virus. The number of libraries from the WWTP after removing diseased and AIV+ birds precludes statistical analysis. Diversity is given by the number of unique genes. Absolute gene abundances were estimated based on a stably expressed host gene.

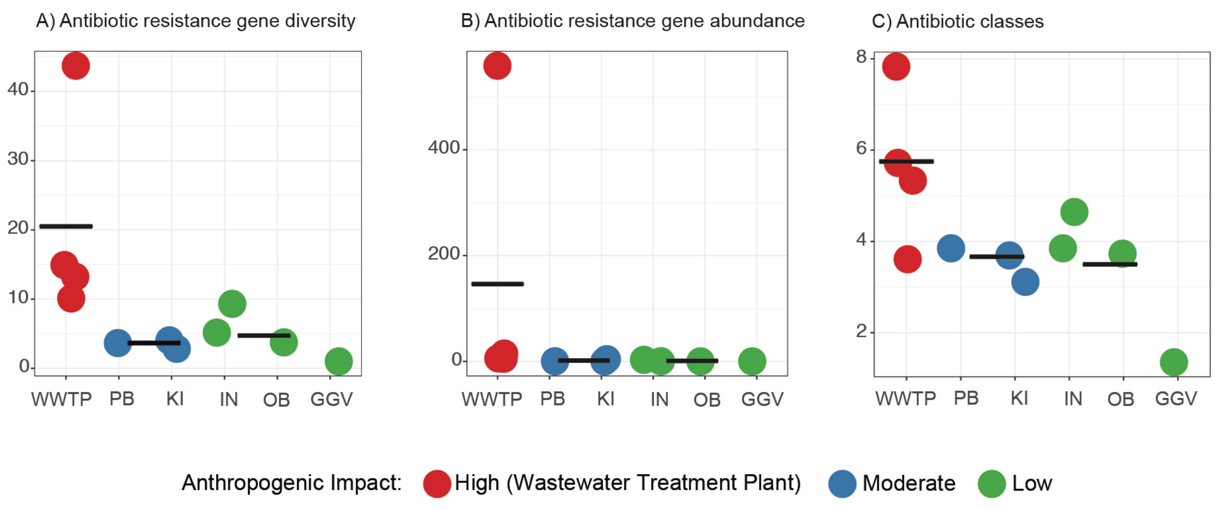

Figure S2. Distribution of antibiotic resistance genes in birds foraging in a wastewater treatment plant (WWTP) compared to birds foraging in sites with moderate and low human impact. Sites were defined as 'low impact' when isolated and/or close to settlements of <50 people, 'moderate impact' when close to cities, small towns or agriculture (considering that agricultural runoffs contain antibiotics), and 'high impact' when sampled at the WWTP (considering that resistant bacteria are only partially removed during water treatment). Each dot represents a meta-transcriptome library (constructed from 10 samples). The number of libraries in each site precludes formal statistical analysis. Black lines indicate the mean values of each category. PB = Western Port Bay, Australia; KI = King Island, Australia; IN = Innamincka Reserve, Australia; OB = O'Higgins Base, Antarctica; GGV = Gabriel González Videla Base, Antarctica.

A) Resistance gene diversity in individual birds from two libraries

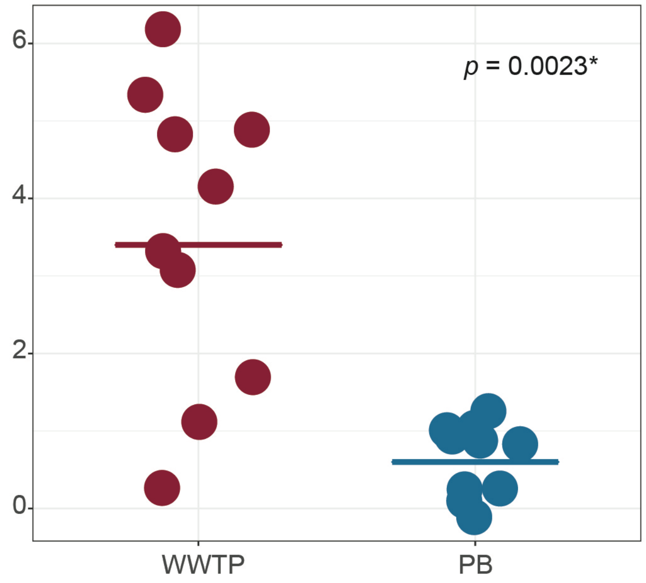

B) Higher prevalence of antibiotic resistance genes in the WWTP

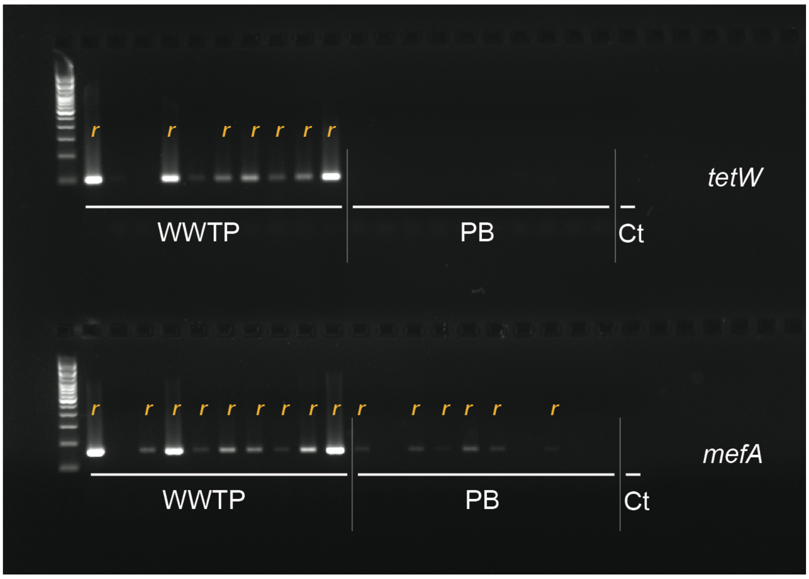

Figure S3. PCR analyses utilizing individual birds confirm that those from the WWTP harbour a higher diversity of antibiotic resistance genes. (A) diversity of resistance genes observed in birds from the wastewater treatment plant (WWTP, n=10) and from Western Port Bay (PB, n=10). Statistical significance was assessed with a Kruskal-Wallis test and differences between WWTP and pristine site were found to be significant (*p*-values < 0.05). (B) PCR results for two resistance genes where the higher prevalence of antibiotic resistance in the WWTP is evident. Ct = negative PCR control. '*r*' indicates amplification of resistance genes.

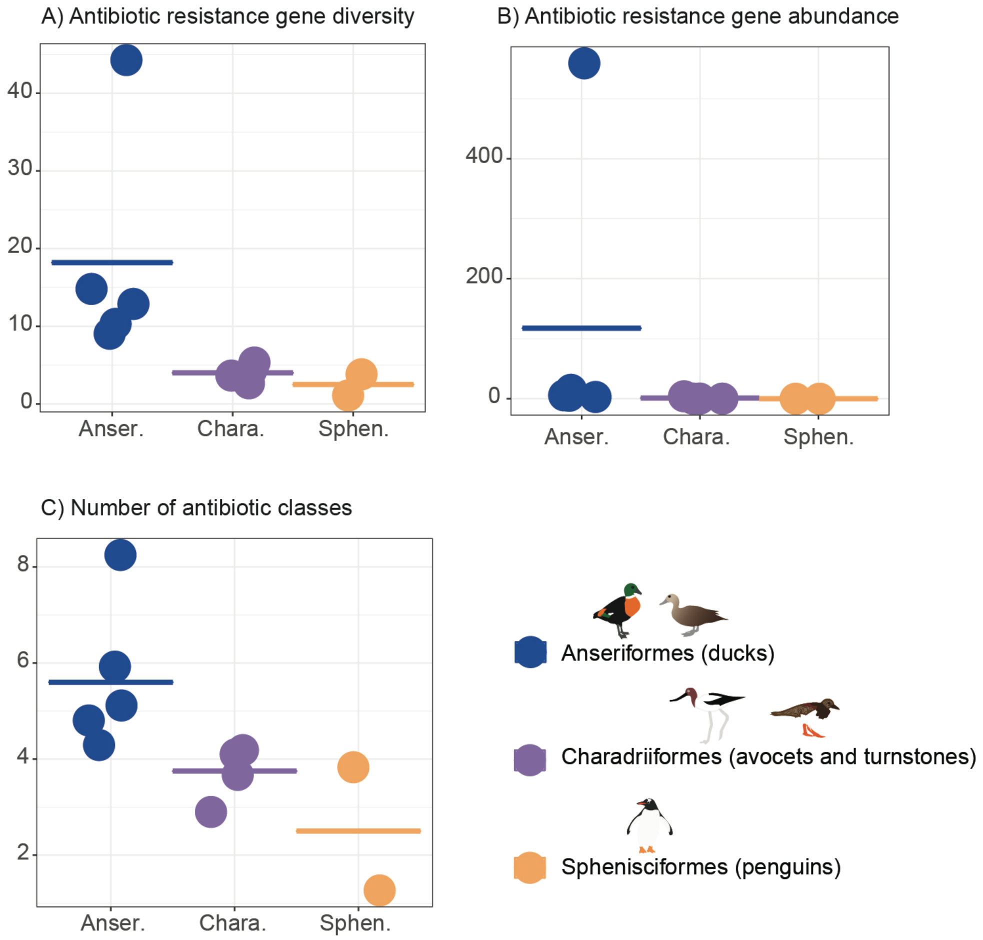

Figure S4. Distribution of antibiotic resistance genes across bird orders. Each dot represents a meta-transcriptome library (constructed from 10 samples) and cross bars represent mean values. The number of penguin libraries precludes statistical analysis.

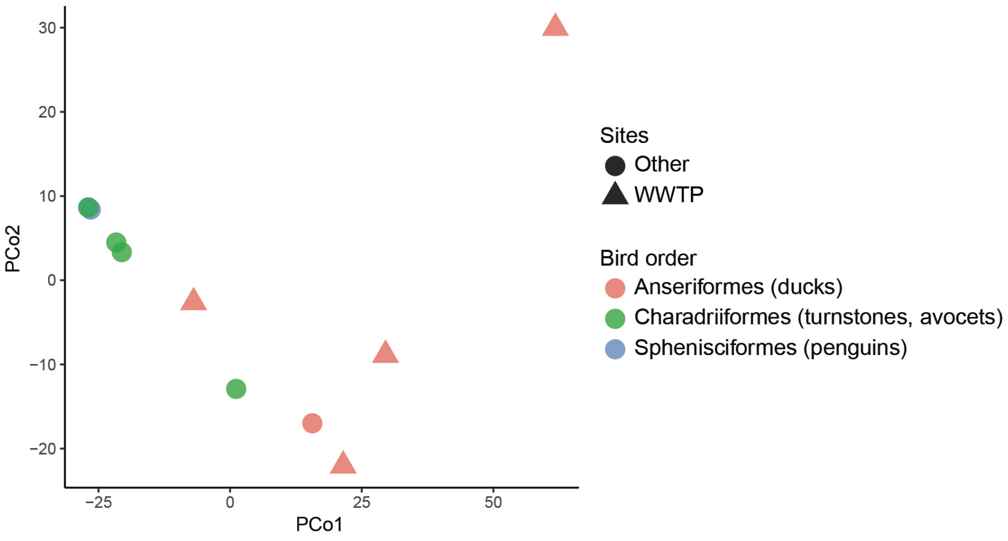

Figure S5. Principal Coordinate Analysis of the expression of metabolic pathways by the microbiome of birds from a wastewater treatment plant (WWTP) and other sites. Colours indicate the bird orders and shapes indicate collection site.

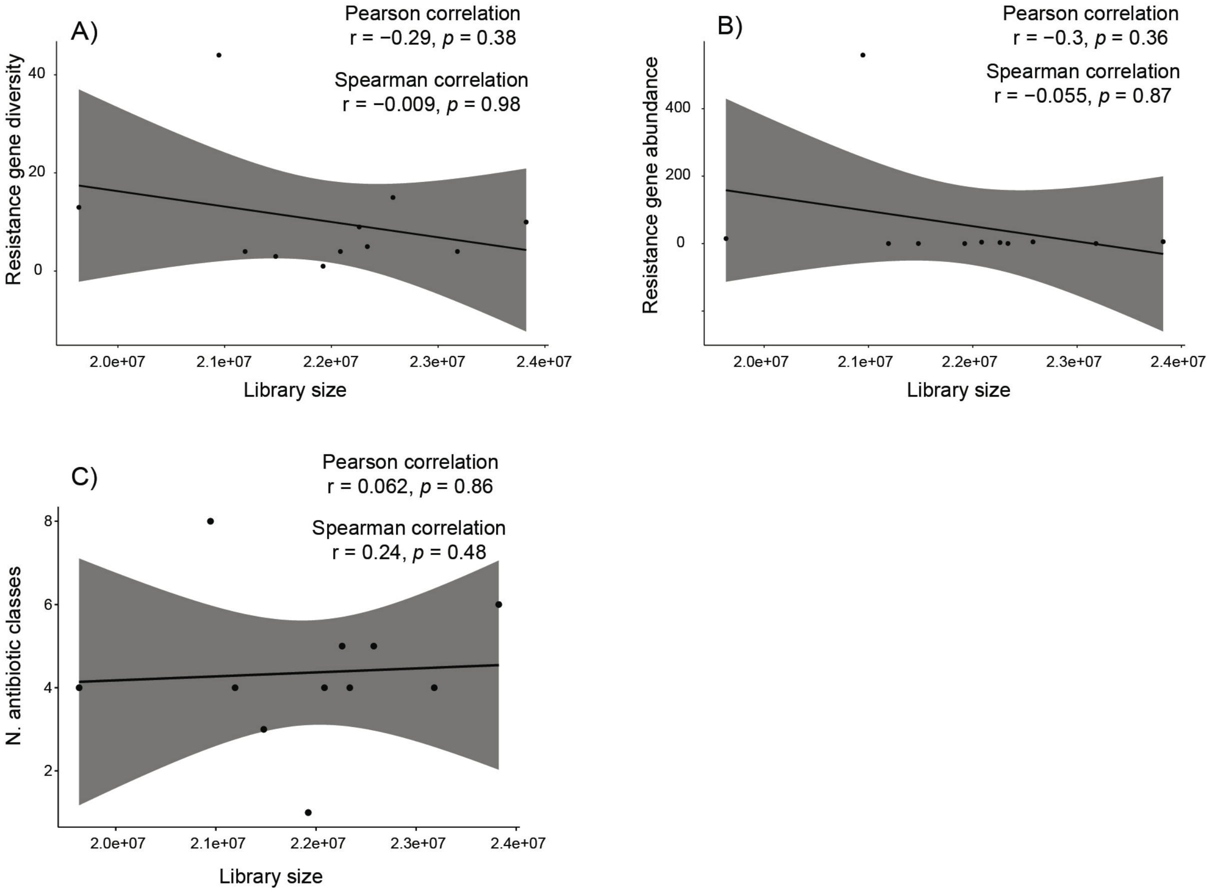

Figure S6. No correlation between library size (sequencing depth) and resistance gene diversity (A), abundance (B) or number of antibiotic classes (C) was observed, indicating that library size does not impact the results. These results indicate that the higher diversity and abundance of resistance genes in ducks from the wastewater treatment plant does not result from unequal sequencing effort. Statistics were performed using Pearson's and Spearman's correlation, and results (non-significant, $p > 0.05$) are given on the top of each graph. Shaded area indicates 95% confidence interval.

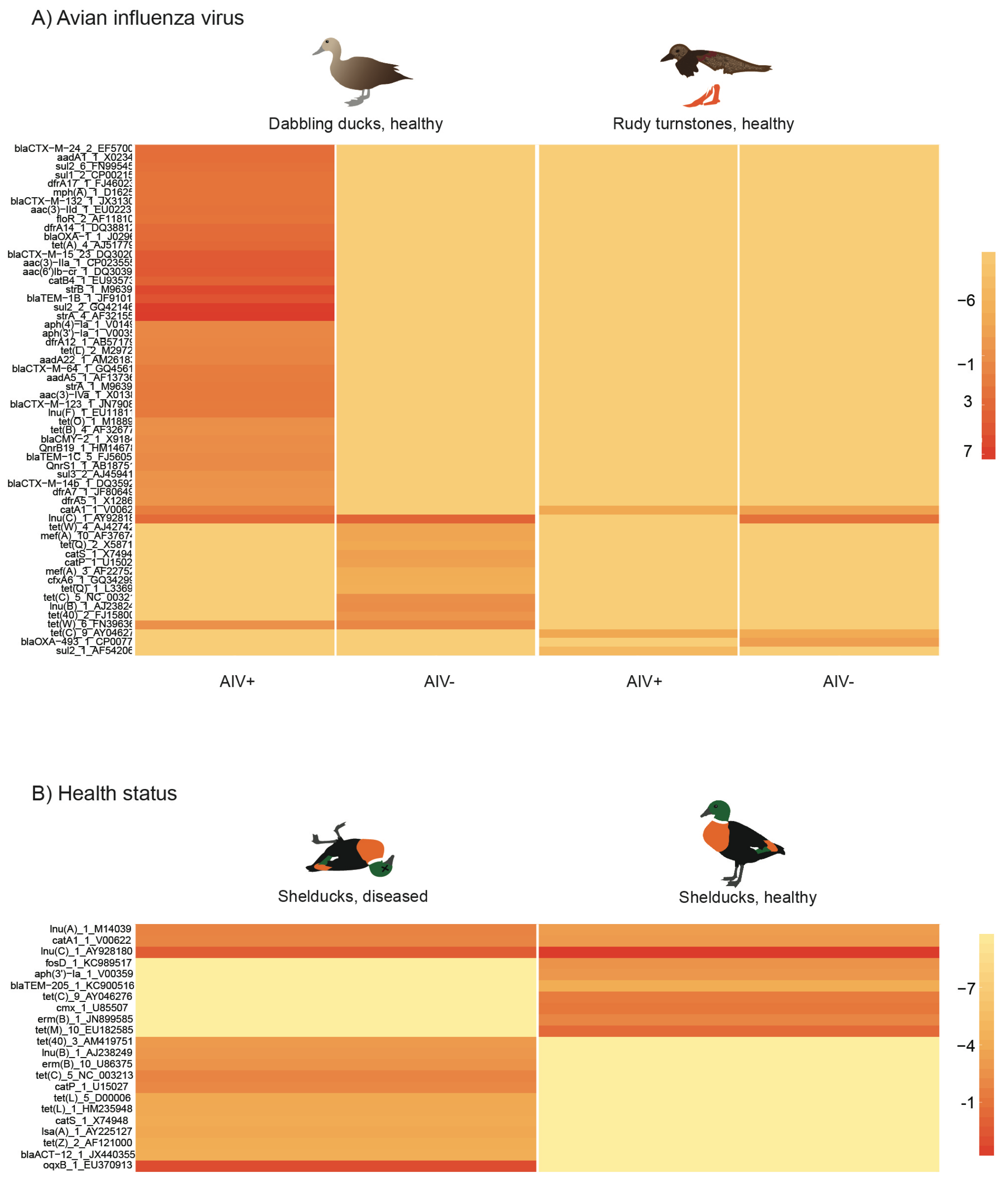

Figure S7. Resistance gene expression profile varies with avian influenza infection (A) and health status (B). Resistance gene names and corresponding NCBI accession numbers are given. Shelduck = Australian shelducks. Estimated gene abundances are represented in $\log_2$ scale. No statistical tests were performed due to the small number of libraries of diseased birds and birds with avian influenza.

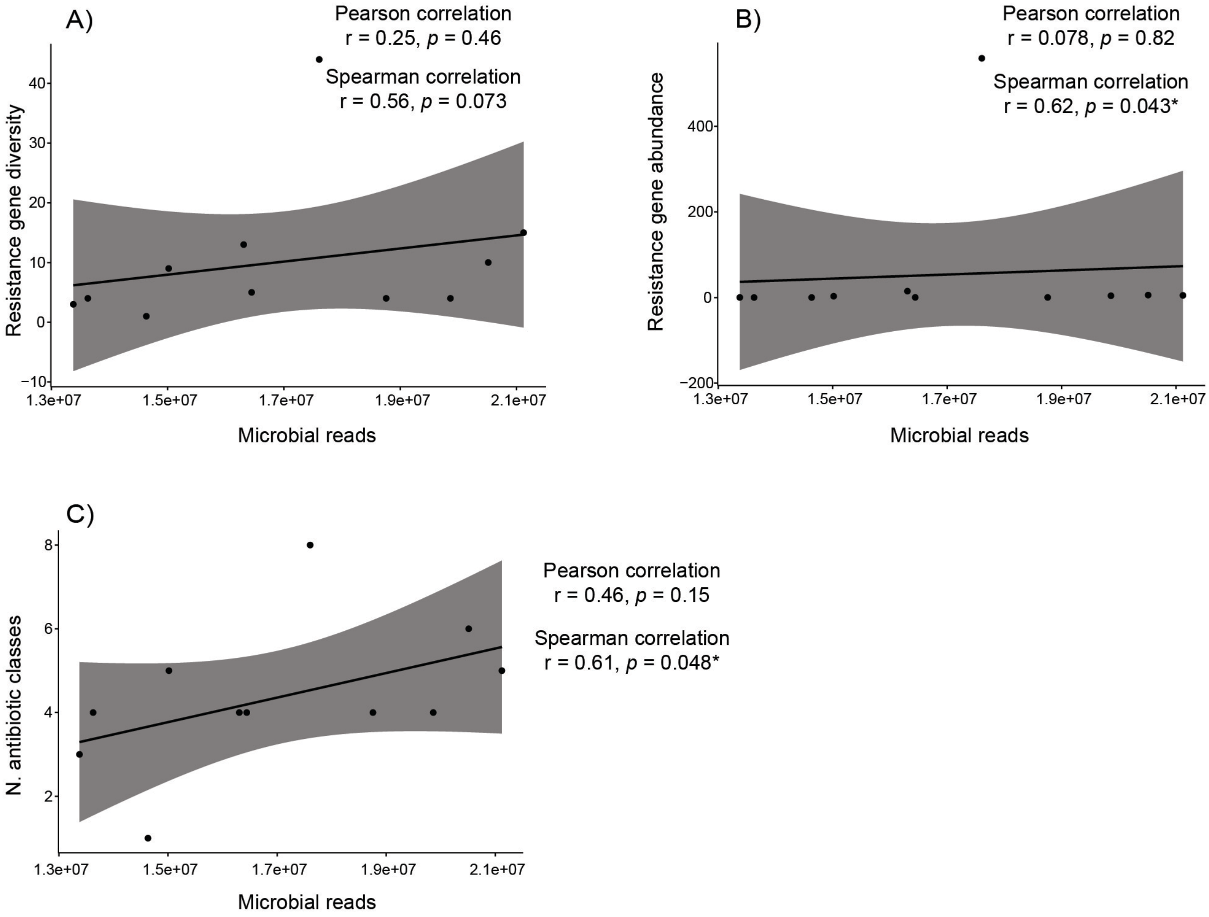

Figure S8. Correlation between the number of reads attributed to the microbiome (after host and rRNA filtering) and the resistance gene burden. Statistics were performed using Pearson's and Spearman's correlation and results are given next to each graph. Shaded area indicates 95% confidence interval. No correlation was observed when using Pearson's test. Resistance gene abundance and number of antibiotic classes were found to correlate significantly (p = 0.04) with number of mRNA reads only when using Spearman's correlation, which is less sensitive to outliers.
